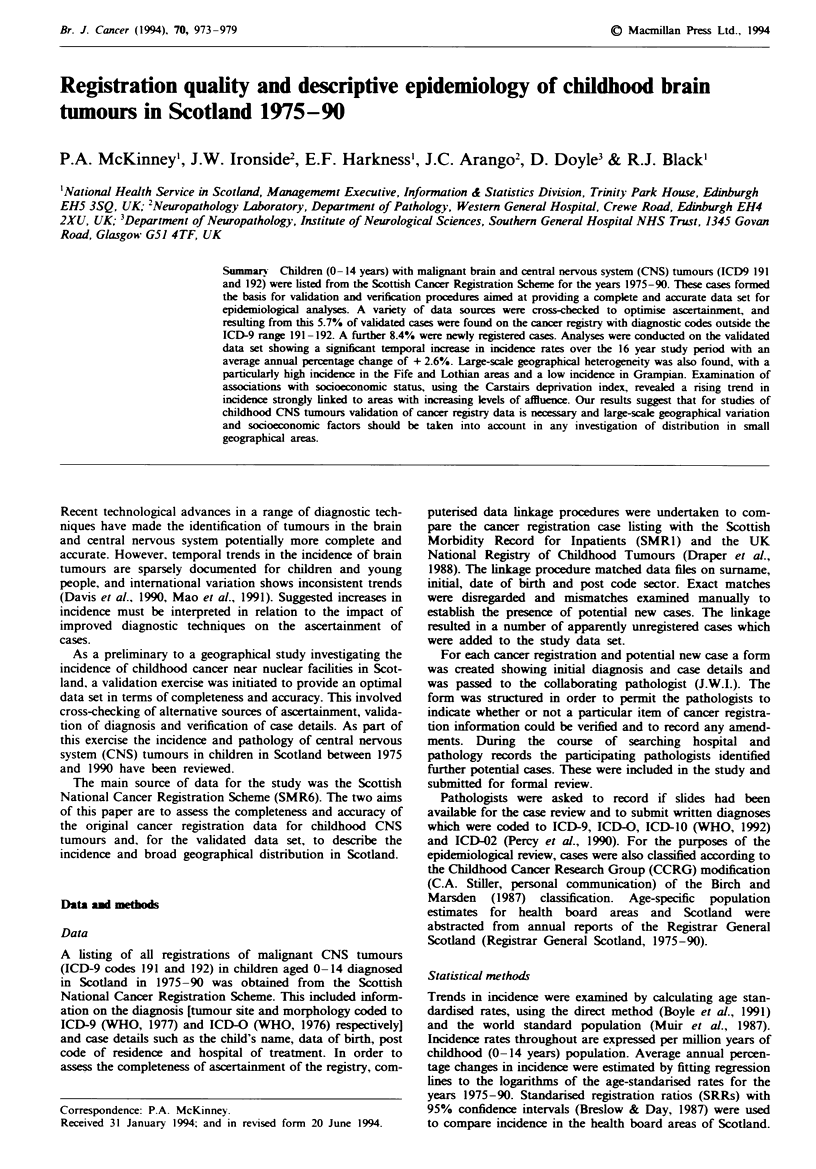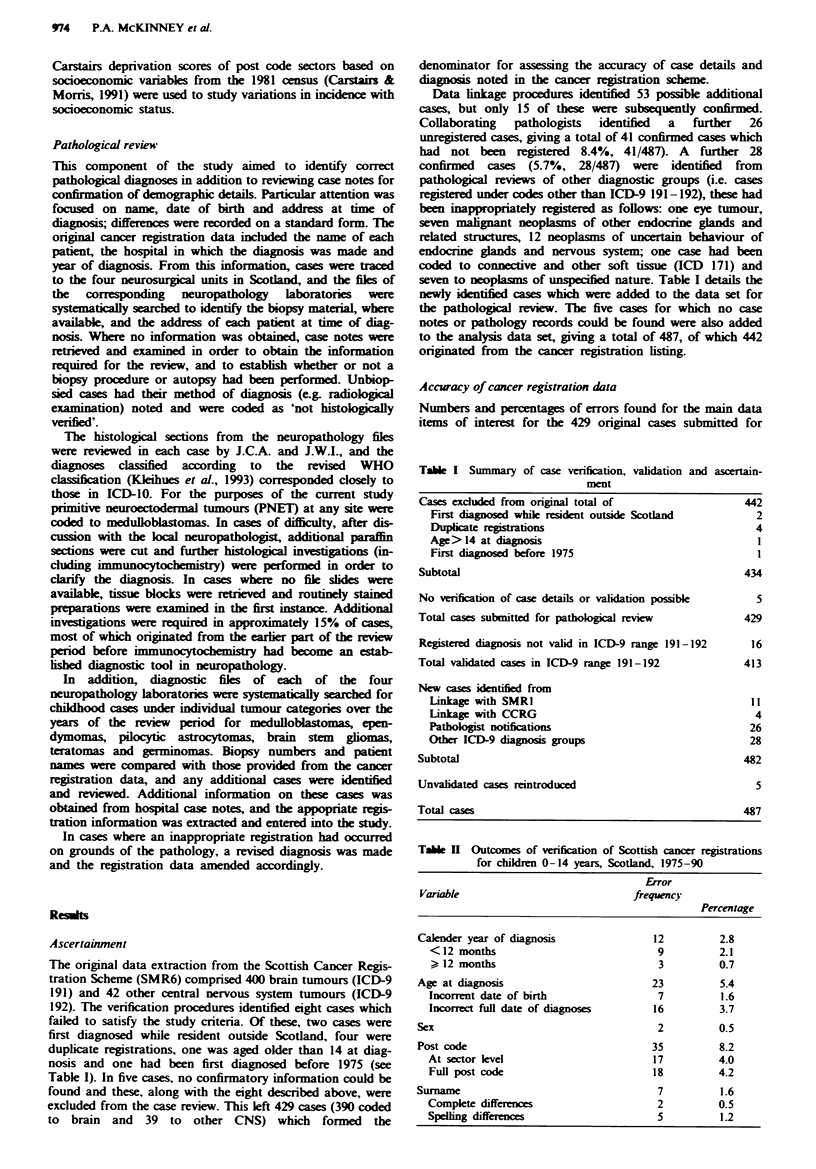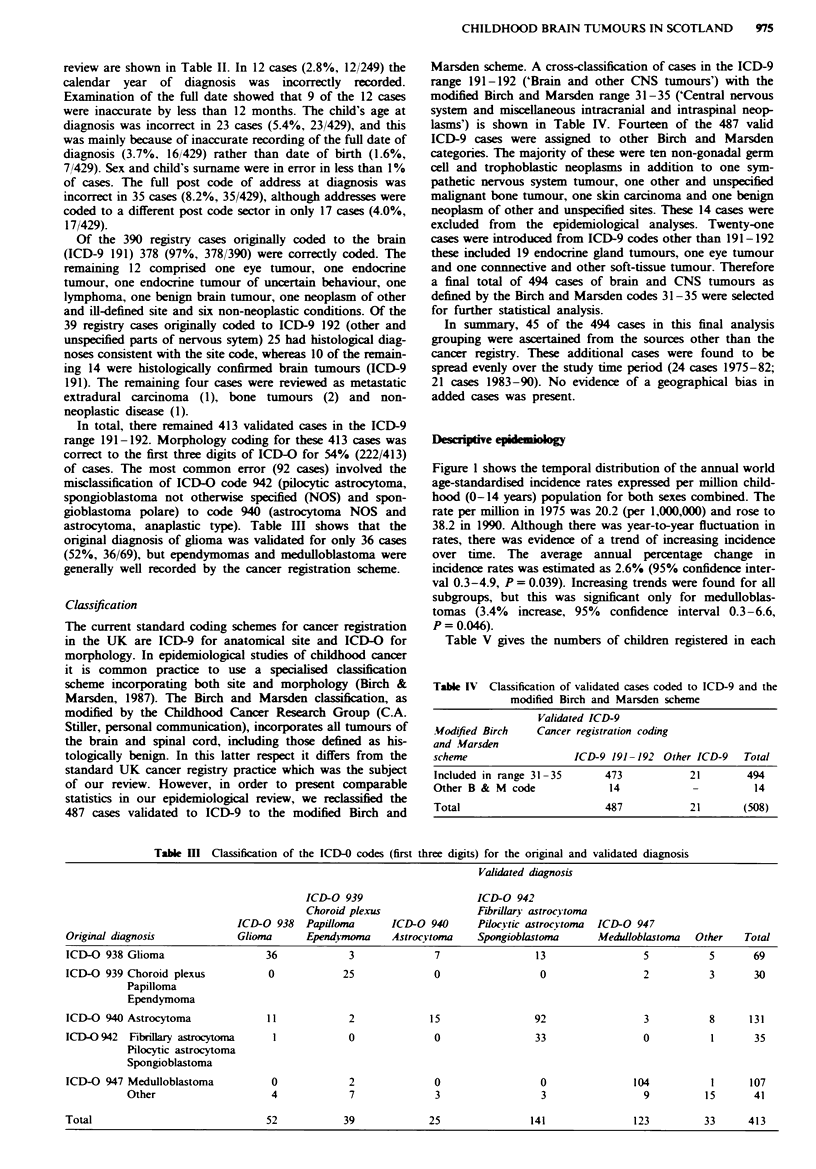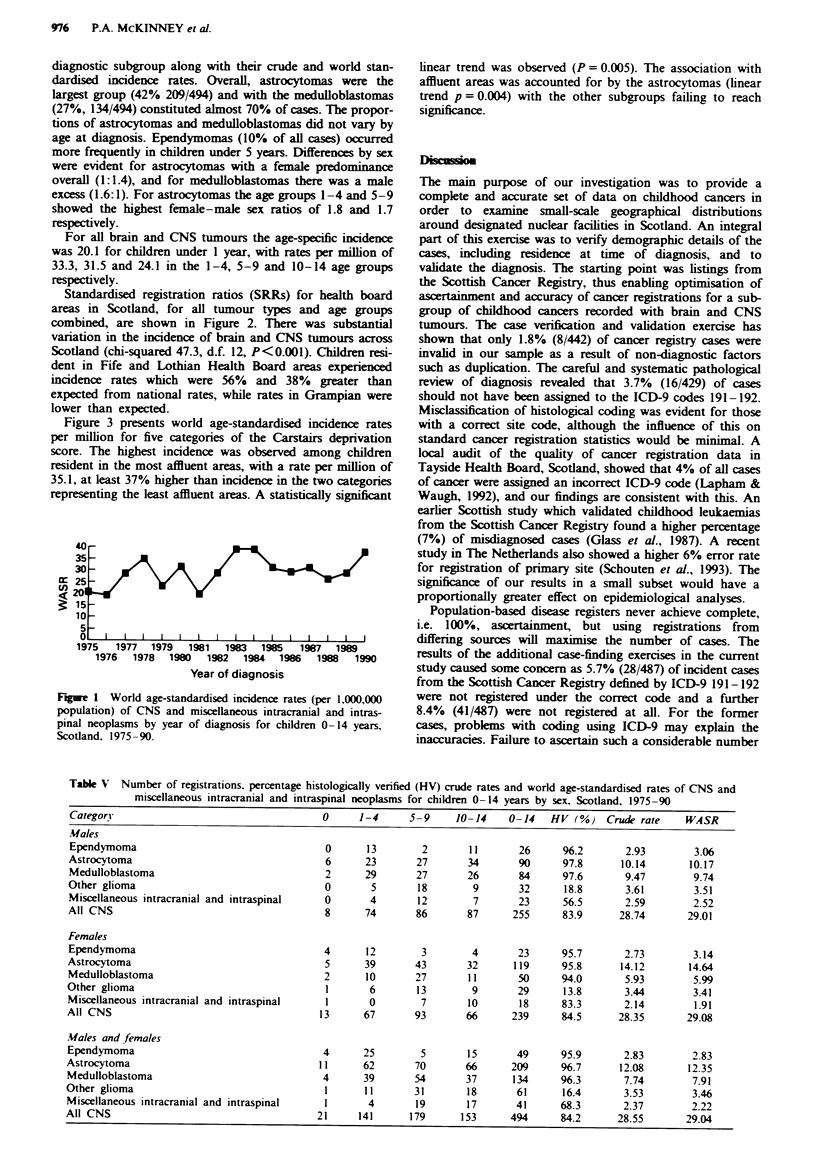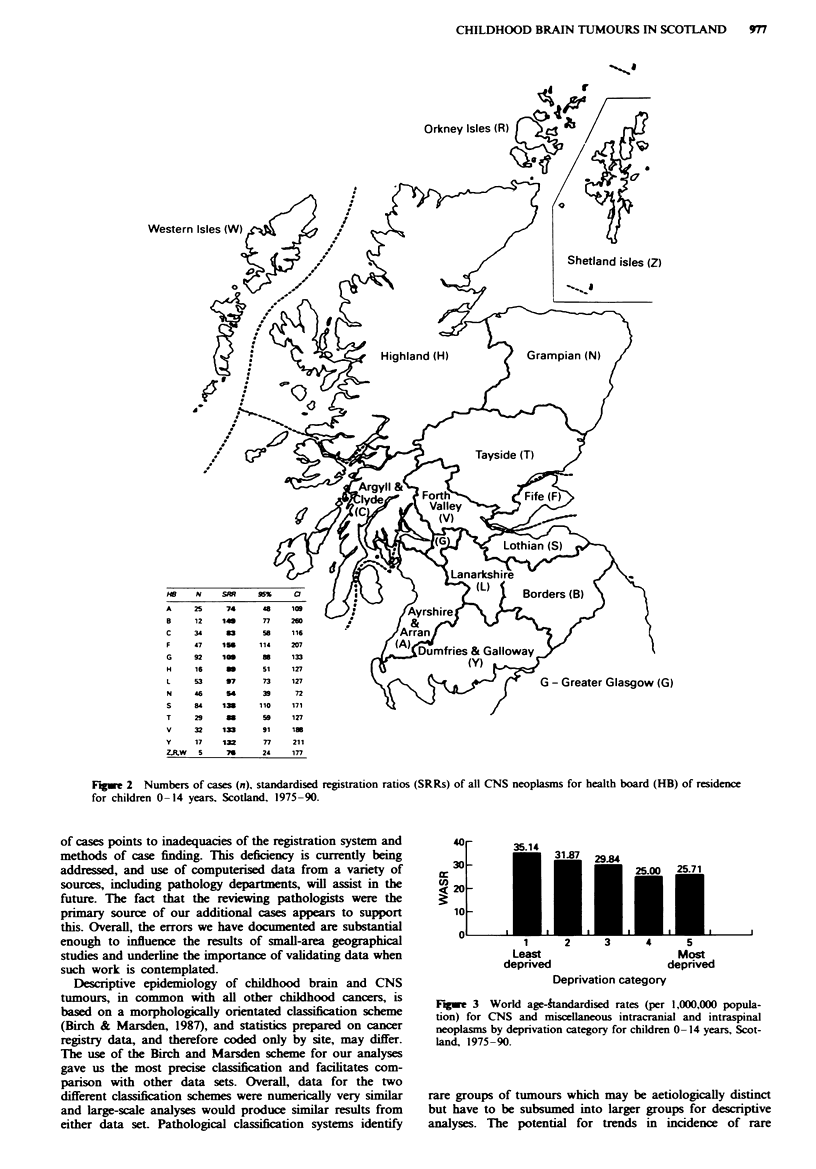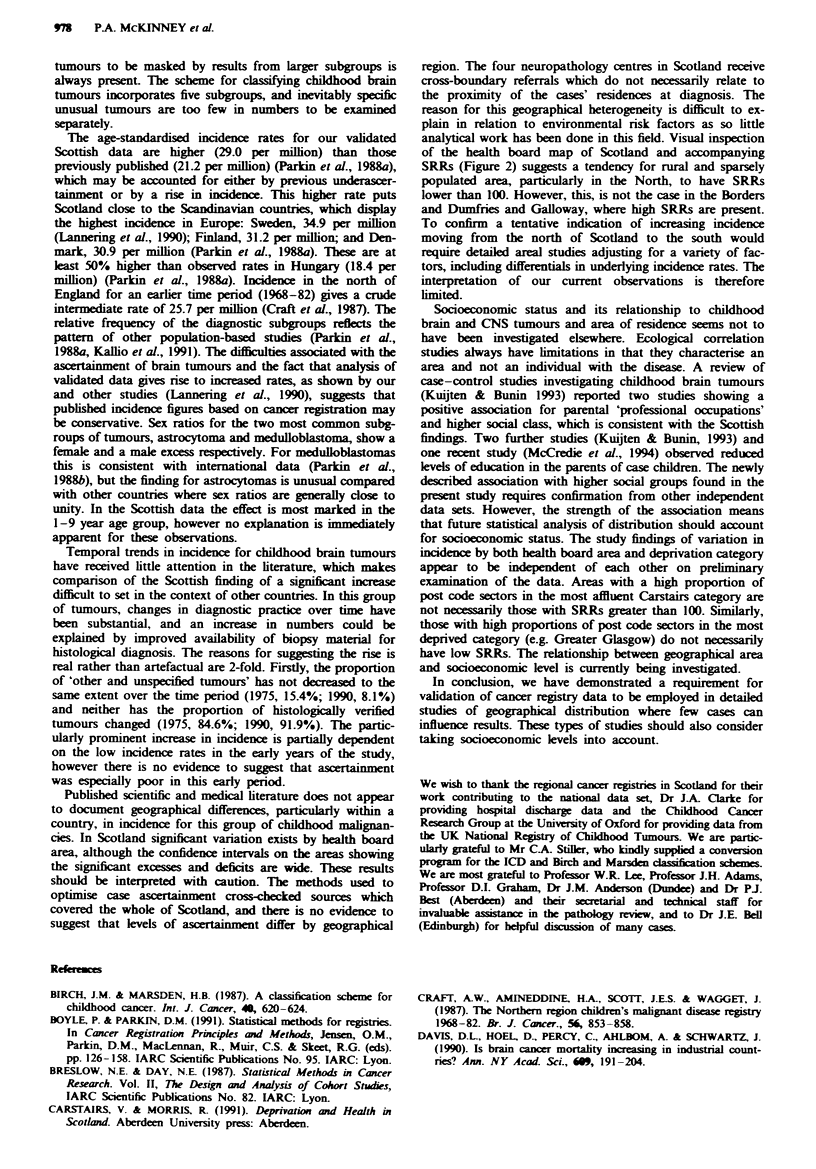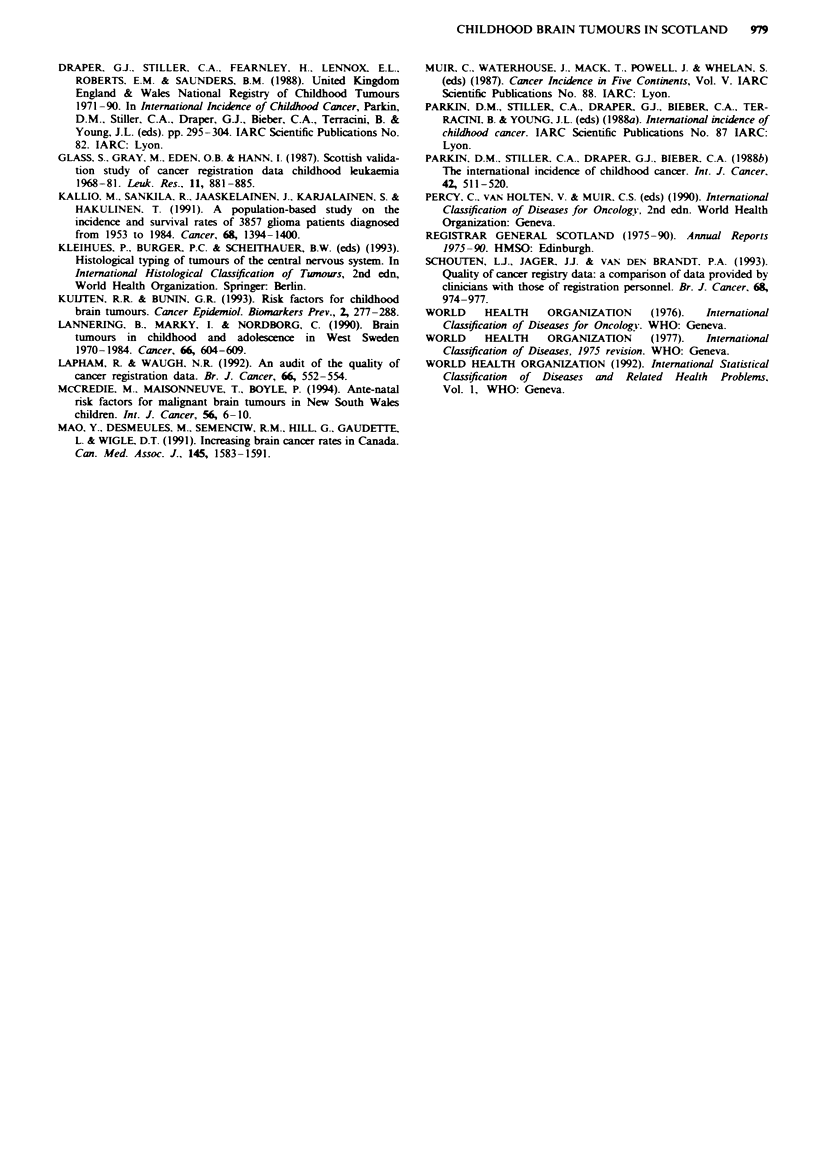# Registration quality and descriptive epidemiology of childhood brain tumours in Scotland 1975-90.

**DOI:** 10.1038/bjc.1994.432

**Published:** 1994-11

**Authors:** P. A. McKinney, J. W. Ironside, E. F. Harkness, J. C. Arango, D. Doyle, R. J. Black

**Affiliations:** National Health Service in Scotland, Management Executive, Information & Statistics Division, Edinburgh, UK.

## Abstract

Children (0-14 years) with malignant brain and central nervous system (CNS) tumours (ICD9 191 and 192) were listed from the Scottish Cancer Registration Scheme for the years 1975-90. These cases formed the basis for validation and verification procedures aimed at providing a complete and accurate data set for epidemiological analyses. A variety of data sources were cross-checked to optimise ascertainment, and resulting from this 5.7% of validated cases were found on the cancer registry with diagnostic codes outside the ICD-9 range 191-192. A further 8.4% were newly registered cases. Analyses were conducted on the validated data set showing a significant temporal increase in incidence rates over the 16 year study period with an average annual percentage change of +2.6%. Large-scale geographical heterogeneity was also found, with a particularly high incidence in the Fife and Lothian areas and a low incidence in Grampian. Examination of associations with socioeconomic status, using the Carstairs deprivation index, revealed a rising trend in incidence strongly linked to areas with increasing levels of affluence. Our results suggest that for studies of childhood CNS tumours validation of cancer registry data is necessary and large-scale geographical variation and socioeconomic factors should be taken into account in any investigation of distribution in small geographical areas.


 © Macmillan Press Ltd., 1994

### Registration quality and descriptive epidemiology of childhood brain tumours in Scotland 1975-90

P.A. McKinney[1], J.W. Ironside[2], E.F. Harkness[1], J.C. Arango[2], D. Doyle[3] & R.J. Black[1]

[1]*National Health Service in Scotland, Managememt Executive, Information & Statistics Division, Trinity Park House, Edinburgh EH5 3SQ, UK;* [2]*Neuropathology Laboratory, Department of Pathology, Western General Hospital, Crewe Road, Edinburgh EH4 2XU, UK;* [3]*Department of Neuropathology, Institute of Neurological Sciences, Southern General Hospital NHS Trust, 1345 Govan Road, Glasgow G51 4TF, UK*

**Summary** Children (0-14 years) with malignant brain and central nervous system (CNS) tumours (ICD9 191 and 192) were listed from the Scottish Cancer Registration Scheme for the years 1975-90. These cases formed the basis for validation and verification procedures aimed at providing a complete and accurate data set for epidemiological analyses. A variety of data sources were cross-checked to optimise ascertainment, and resulting from this 5.7% of validated cases were found on the cancer registry with diagnostic codes outside the ICD-9 range 191-192. A further 8.4% were newly registered cases. Analyses were conducted on the validated data set showing a significant temporal increase in incidence rates over the 16 year study period with an average annual percentage change of + 2.6%. Large-scale geographical heterogeneity was also found, with a particularly high incidence in the Fife and Lothian areas and a low incidence in Grampian. Examination of associations with socioeconomic status, using the Carstairs deprivation index, revealed a rising trend in incidence strongly linked to areas with increasing levels of affluence. Our results suggest that for studies of childhood CNS tumours validation of cancer registry data is necessary and large-scale geographical variation and socioeconomic factors should be taken into account in any investigation of distribution in small geographical areas.

Recent technological advances in a range of diagnostic techniques have made the identification of tumours in the brain and central nervous system potentially more complete and accurate. However, temporal trends in the incidence of brain tumours are sparsely documented for children and young people, and international variation shows inconsistent trends (Davis *et al.*, 1990, Mao *et al.*, 1991). Suggested increases in incidence must be interpreted in relation to the impact of improved diagnostic techniques on the ascertainment of cases.

As a preliminary to a geographical study investigating the incidence of childhood cancer near nuclear facilities in Scotland, a validation exercise was initiated to provide an optimal data set in terms of completeness and accuracy. This involved cross-checking of alternative sources of ascertainment, validation of diagnosis and verification of case details. As part of this exercise the incidence and pathology of central nervous system (CNS) tumours in children in Scotland between 1975 and 1990 have been reviewed.

The main source of data for the study was the Scottish National Cancer Registration Scheme (SMR6). The two aims of this paper are to assess the completeness and accuracy of the original cancer registration data for childhood CNS tumours and, for the validated data set, to describe the incidence and broad geographical distribution in Scotland.

### Data and methods

### *Data*

A listing of all registrations of malignant CNS tumours (ICD-9 codes 191 and 192) in children aged 0-14 diagnosed in Scotland in 1975-90 was obtained from the Scottish National Cancer Registration Scheme. This included information on the diagnosis [tumour site and morphology coded to ICD-9 (WHO, 1977) and ICD-O (WHO, 1976) respectively] and case details such as the child's name, data of birth, post code of residence and hospital of treatment. In order to assess the completeness of ascertainment of the registry, computerised data linkage procedures were undertaken to compare the cancer registration case listing with the Scottish Morbidity Record for Inpatients (SMR1) and the UK National Registry of Childhood Tumours (Draper *et al.*, 1988). The linkage procedure matched data files on surname, initial, date of birth and post code sector. Exact matches were disregarded and mismatches examined manually to establish the presence of potential new cases. The linkage resulted in a number of apparently unregistered cases which were added to the study data set.

For each cancer registration and potential new case a form was created showing initial diagnosis and case details and was passed to the collaborating pathologist (J.W.I.). The form was structured in order to permit the pathologists to indicate whether or not a particular item of cancer registration information could be verified and to record any amendments. During the course of searching hospital and pathology records the participating pathologists identified further potential cases. These were included in the study and submitted for formal review.

Pathologists were asked to record if slides had been available for the case review and to submit written diagnoses which were coded to ICD-9, ICD-O, ICD-10 (WHO, 1992) and ICD-02 (Percy *et al.*, 1990). For the purposes of the epidemiological review, cases were also classified according to the Childhood Cancer Research Group (CCRG) modification (C.A. Stiller, personal communication) of the Birch and Marsden (1987) classification. Age-specific population estimates for health board areas and Scotland were abstracted from annual reports of the Registrar General Scotland (Registrar General Scotland, 1975-90).

### *Statistical methods*

Trends in incidence were examined by calculating age standardised rates, using the direct method (Boyle *et al.*, 1991) and the world standard population (Muir *et al.*, 1987). Incidence rates throughout are expressed per million years of childhood (0-14 years) population. Average annual percentage changes in incidence were estimated by fitting regression lines to the logarithms of the age-standarised rates for the years 1975-90. Standarised registration ratios (SRRs) with 95% confidence intervals (Breslow & Day, 1987) were used to compare incidence in the health board areas of Scotland.

Correspondence: P.A. McKinney.
Received 31 January 1994; and in revised form 20 June 1994.

Carstairs deprivation scores of post code sectors based on socioeconomic variables from the 1981 census (Carstairs & Morris, 1991) were used to study variations in incidence with socioeconomic status.

*Pathological review*

This component of the study aimed to identify correct pathological diagnoses in addition to reviewing case notes for confirmation of demographic details. Particular attention was focused on name, date of birth and address at time of diagnosis; differences were recorded on a standard form. The original cancer registration data included the name of each patient, the hospital in which the diagnosis was made and year of diagnosis. From this information, cases were traced to the four neurosurgical units in Scotland, and the files of the corresponding neuropathology laboratories were systematically searched to identify the biopsy material, where available, and the address of each patient at time of diagnosis. Where no information was obtained, case notes were retrieved and examined in order to obtain the information required for the review, and to establish whether or not a biopsy procedure or autopsy had been performed. Unbiopsied cases had their method of diagnosis (e.g. radiological examination) noted and were coded as 'not histologically verified'.

The histological sections from the neuropathology files were reviewed in each case by J.C.A. and J.W.I., and the diagnoses classified according to the revised WHO classification (Kleihues *et al.*, 1993) corresponded closely to those in ICD-10. For the purposes of the current study primitive neuroectodermal tumours (PNET) at any site were coded to medulloblastomas. In cases of difficulty, after discussion with the local neuropathologist, additional paraffin sections were cut and further histological investigations (including immunocytochemistry) were performed in order to clarify the diagnosis. In cases where no file slides were available, tissue blocks were retrieved and routinely stained preparations were examined in the first instance. Additional investigations were required in approximately 15% of cases, most of which originated from the earlier part of the review period before immunocytochemistry had become an established diagnostic tool in neuropathology.

In addition, diagnostic files of each of the four neuropathology laboratories were systematically searched for childhood cases under individual tumour categories over the years of the review period for medulloblastomas, ependymomas, pilocytic astrocytomas, brain stem gliomas, teratomas and germinomas. Biopsy numbers and patient names were compared with those provided from the cancer registration data, and any additional cases were identified and reviewed. Additional information on these cases was obtained from hospital case notes, and the appopriate registration information was extracted and entered into the study.

In cases where an inappropriate registration had occurred on grounds of the pathology, a revised diagnosis was made and the registration data amended accordingly.

### Results

*Ascertainment*

The original data extraction from the Scottish Cancer Registration Scheme (SMR6) comprised 400 brain tumours (ICD-9 191) and 42 other central nervous system tumours (ICD-9 192). The verification procedures identified eight cases which failed to satisfy the study criteria. Of these, two cases were first diagnosed while resident outside Scotland, four were duplicate registrations, one was aged older than 14 at diagnosis and one had been first diagnosed before 1975 (see Table I). In five cases, no confirmatory information could be found and these, along with the eight described above, were excluded from the case review. This left 429 cases (390 coded to brain and 39 to other CNS) which formed the denominator for assessing the accuracy of case details and diagnosis noted in the cancer registration scheme.

Data linkage procedures identified 53 possible additional cases, but only 15 of these were subsequently confirmed. Collaborating pathologists identified a further 26 unregistered cases, giving a total of 41 confirmed cases which had not been registered 8.4%, 41/487). A further 28 confirmed cases (5.7%, 28/487) were identified from pathological reviews of other diagnostic groups (i.e. cases registered under codes other than ICD-9 191-192), these had been inappropriately registered as follows: one eye tumour, seven malignant neoplasms of other endocrine glands and related structures, 12 neoplasms of uncertain behaviour of endocrine glands and nervous system; one case had been coded to connective and other soft tissue (ICD 171) and seven to neoplasms of unspecified nature. Table I details the newly identified cases which were added to the data set for the pathological review. The five cases for which no case notes or pathology records could be found were also added to the analysis data set, giving a total of 487, of which 442 originated from the cancer registration listing.

*Accuracy of cancer registration data*

Numbers and percentages of errors found for the main data items of interest for the 429 original cases submitted for

**Table I** Summary of case verification, validation and ascertainment

| | |
|---|---|
| Cases excluded from original total of | 442 |
| First diagnosed while resident outside Scotland | 2 |
| Duplicate registrations | 4 |
| Age > 14 at diagnosis | 1 |
| First diagnosed before 1975 | 1 |
| Subtotal | 434 |
| No verification of case details or validation possible | 5 |
| Total cases submitted for pathological review | 429 |
| Registered diagnosis not valid in ICD-9 range 191-192 | 16 |
| Total validated cases in ICD-9 range 191-192 | 413 |
| New cases identified from | |
| Linkage with SMR1 | 11 |
| Linkage with CCRG | 4 |
| Pathologist notifications | 26 |
| Other ICD-9 diagnosis groups | 28 |
| Subtotal | 482 |
| Unvalidated cases reintroduced | 5 |
| Total cases | 487 |

**Table II** Outcomes of verification of Scottish cancer registrations for children 0-14 years, Scotland, 1975-90

| *Variable* | *Error frequency* | *Percentage* |
|---|---|---|
| Calender year of diagnosis | 12 | 2.8 |
| < 12 months | 9 | 2.1 |
| ≥ 12 months | 3 | 0.7 |
| Age at diagnosis | 23 | 5.4 |
| Incorrent date of birth | 7 | 1.6 |
| Incorrect full date of diagnoses | 16 | 3.7 |
| Sex | 2 | 0.5 |
| Post code | 35 | 8.2 |
| At sector level | 17 | 4.0 |
| Full post code | 18 | 4.2 |
| Surname | 7 | 1.6 |
| Complete differences | 2 | 0.5 |
| Spelling differences | 5 | 1.2 |

review are shown in Table II. In 12 cases (2.8%, 12/249) the calendar year of diagnosis was incorrectly recorded. Examination of the full date showed that 9 of the 12 cases were inaccurate by less than 12 months. The child's age at diagnosis was incorrect in 23 cases (5.4%, 23/429), and this was mainly because of inaccurate recording of the full date of diagnosis (3.7%, 16/429) rather than date of birth (1.6%, 7/429). Sex and child's surname were in error in less than 1% of cases. The full post code of address at diagnosis was incorrect in 35 cases (8.2%, 35/429), although addresses were coded to a different post code sector in only 17 cases (4.0%, 17/429).

Of the 390 registry cases originally coded to the brain (ICD-9 191) 378 (97%, 378/390) were correctly coded. The remaining 12 comprised one eye tumour, one endocrine tumour, one endocrine tumour of uncertain behaviour, one lymphoma, one benign brain tumour, one neoplasm of other and ill-defined site and six non-neoplastic conditions. Of the 39 registry cases originally coded to ICD-9 192 (other and unspecified parts of nervous sytem) 25 had histological diagnoses consistent with the site code, whereas 10 of the remaining 14 were histologically confirmed brain tumours (ICD-9 191). The remaining four cases were reviewed as metastatic extradural carcinoma (1), bone tumours (2) and non-neoplastic disease (1).

In total, there remained 413 validated cases in the ICD-9 range 191–192. Morphology coding for these 413 cases was correct to the first three digits of ICD-O for 54% (222/413) of cases. The most common error (92 cases) involved the misclassification of ICD-O code 942 (pilocytic astrocytoma, spongioblastoma not otherwise specified (NOS) and spongioblastoma polare) to code 940 (astrocytoma NOS and astrocytoma, anaplastic type). Table III shows that the original diagnosis of glioma was validated for only 36 cases (52%, 36/69), but ependymomas and medulloblastoma were generally well recorded by the cancer registration scheme.

*Classification*

The current standard coding schemes for cancer registration in the UK are ICD-9 for anatomical site and ICD-O for morphology. In epidemiological studies of childhood cancer it is common practice to use a specialised classification scheme incorporating both site and morphology (Birch & Marsden, 1987). The Birch and Marsden classification, as modified by the Childhood Cancer Research Group (C.A. Stiller, personal communication), incorporates all tumours of the brain and spinal cord, including those defined as histologically benign. In this latter respect it differs from the standard UK cancer registry practice which was the subject of our review. However, in order to present comparable statistics in our epidemiological review, we reclassified the 487 cases validated to ICD-9 to the modified Birch and Marsden scheme. A cross-classification of cases in the ICD-9 range 191–192 ('Brain and other CNS tumours') with the modified Birch and Marsden range 31–35 ('Central nervous system and miscellaneous intracranial and intraspinal neoplasms') is shown in Table IV. Fourteen of the 487 valid ICD-9 cases were assigned to other Birch and Marsden categories. The majority of these were ten non-gonadal germ cell and trophoblastic neoplasms in addition to one sympathetic nervous system tumour, one other and unspecified malignant bone tumour, one skin carcinoma and one benign neoplasm of other and unspecified sites. These 14 cases were excluded from the epidemiological analyses. Twenty-one cases were introduced from ICD-9 codes other than 191–192 these included 19 endocrine gland tumours, one eye tumour and one connnective and other soft-tissue tumour. Therefore a final total of 494 cases of brain and CNS tumours as defined by the Birch and Marsden codes 31–35 were selected for further statistical analysis.

In summary, 45 of the 494 cases in this final analysis grouping were ascertained from the sources other than the cancer registry. These additional cases were found to be spread evenly over the study time period (24 cases 1975–82; 21 cases 1983–90). No evidence of a geographical bias in added cases was present.

**Descriptive epidemiology**

Figure 1 shows the temporal distribution of the annual world age-standardised incidence rates expressed per million childhood (0–14 years) population for both sexes combined. The rate per million in 1975 was 20.2 (per 1,000,000) and rose to 38.2 in 1990. Although there was year-to-year fluctuation in rates, there was evidence of a trend of increasing incidence over time. The average annual percentage change in incidence rates was estimated as 2.6% (95% confidence interval 0.3–4.9, $P = 0.039$). Increasing trends were found for all subgroups, but this was significant only for medulloblastomas (3.4% increase, 95% confidence interval 0.3–6.6, $P = 0.046$).

Table V gives the numbers of children registered in each

**Table IV** Classification of validated cases coded to ICD-9 and the modified Birch and Marsden scheme

| *Modified Birch and Marsden scheme* | *Validated ICD-9 Cancer registration coding* ICD-9 191–192 | *Other ICD-9* | *Total* |
|---|---|---|---|
| Included in range 31–35 | 473 | 21 | 494 |
| Other B & M code | 14 | – | 14 |
| Total | 487 | 21 | (508) |

**Table III** Classification of the ICD-0 codes (first three digits) for the original and validated diagnosis

| | *Validated diagnosis* | | | | | | |
|---|---|---|---|---|---|---|---|
| *Original diagnosis* | *ICD-O 938 Glioma* | *ICD-O 939 Choroid plexus Papilloma Ependymoma* | *ICD-O 940 Astrocytoma* | *ICD-O 942 Fibrillary astrocytoma Pilocytic astrocytoma Spongioblastoma* | *ICD-O 947 Medulloblastoma* | *Other* | *Total* |
| ICD-O 938 Glioma | 36 | 3 | 7 | 13 | 5 | 5 | 69 |
| ICD-O 939 Choroid plexus Papilloma Ependymoma | 0 | 25 | 0 | 0 | 2 | 3 | 30 |
| ICD-O 940 Astrocytoma | 11 | 2 | 15 | 92 | 3 | 8 | 131 |
| ICD-O 942 Fibrillary astrocytoma Pilocytic astrocytoma Spongioblastoma | 1 | 0 | 0 | 33 | 0 | 1 | 35 |
| ICD-O 947 Medulloblastoma | 0 | 2 | 0 | 0 | 104 | 1 | 107 |
| Other | 4 | 7 | 3 | 3 | 9 | 15 | 41 |
| Total | 52 | 39 | 25 | 141 | 123 | 33 | 413 |

diagnostic subgroup along with their crude and world standardised incidence rates. Overall, astrocytomas were the largest group (42% 209/494) and with the medulloblastomas (27%, 134/494) constituted almost 70% of cases. The proportions of astrocytomas and medulloblastomas did not vary by age at diagnosis. Ependymomas (10% of all cases) occurred more frequently in children under 5 years. Differences by sex were evident for astrocytomas with a female predominance overall (1:1.4), and for medulloblastomas there was a male excess (1.6:1). For astrocytomas the age groups 1–4 and 5–9 showed the highest female–male sex ratios of 1.8 and 1.7 respectively.

For all brain and CNS tumours the age-specific incidence was 20.1 for children under 1 year, with rates per million of 33.3, 31.5 and 24.1 in the 1–4, 5–9 and 10–14 age groups respectively.

Standardised registration ratios (SRRs) for health board areas in Scotland, for all tumour types and age groups combined, are shown in Figure 2. There was substantial variation in the incidence of brain and CNS tumours across Scotland (chi-squared 47.3, d.f. 12, $P<0.001$). Children resident in Fife and Lothian Health Board areas experienced incidence rates which were 56% and 38% greater than expected from national rates, while rates in Grampian were lower than expected.

Figure 3 presents world age-standardised incidence rates per million for five categories of the Carstairs deprivation score. The highest incidence was observed among children resident in the most affluent areas, with a rate per million of 35.1, at least 37% higher than incidence in the two categories representing the least affluent areas. A statistically significant

**Figure 1** World age-standardised incidence rates (per 1,000,000 population) of CNS and miscellaneous intracranial and intraspinal neoplasms by year of diagnosis for children 0–14 years, Scotland, 1975–90.

linear trend was observed ($P = 0.005$). The association with affluent areas was accounted for by the astrocytomas (linear trend $p = 0.004$) with the other subgroups failing to reach significance.

### Discussion

The main purpose of our investigation was to provide a complete and accurate set of data on childhood cancers in order to examine small-scale geographical distributions around designated nuclear facilities in Scotland. An integral part of this exercise was to verify demographic details of the cases, including residence at time of diagnosis, and to validate the diagnosis. The starting point was listings from the Scottish Cancer Registry, thus enabling optimisation of ascertainment and accuracy of cancer registrations for a subgroup of childhood cancers recorded with brain and CNS tumours. The case verification and validation exercise has shown that only 1.8% (8/442) of cancer registry cases were invalid in our sample as a result of non-diagnostic factors such as duplication. The careful and systematic pathological review of diagnosis revealed that 3.7% (16/429) of cases should not have been assigned to the ICD-9 codes 191–192. Misclassification of histological coding was evident for those with a correct site code, although the influence of this on standard cancer registration statistics would be minimal. A local audit of the quality of cancer registration data in Tayside Health Board, Scotland, showed that 4% of all cases of cancer were assigned an incorrect ICD-9 code (Lapham & Waugh, 1992), and our findings are consistent with this. An earlier Scottish study which validated childhood leukaemias from the Scottish Cancer Registry found a higher percentage (7%) of misdiagnosed cases (Glass et al., 1987). A recent study in The Netherlands also showed a higher 6% error rate for registration of primary site (Schouten et al., 1993). The significance of our results in a small subset would have a proportionally greater effect on epidemiological analyses.

Population-based disease registers never achieve complete, i.e. 100%, ascertainment, but using registrations from differing sources will maximise the number of cases. The results of the additional case-finding exercises in the current study caused some concern as 5.7% (28/487) of incident cases from the Scottish Cancer Registry defined by ICD-9 191–192 were not registered under the correct code and a further 8.4% (41/487) were not registered at all. For the former cases, problems with coding using ICD-9 may explain the inaccuracies. Failure to ascertain such a considerable number

**Table V** Number of registrations, percentage histologically verified (HV) crude rates and world age-standardised rates of CNS and miscellaneous intracranial and intraspinal neoplasms for children 0–14 years by sex, Scotland, 1975–90

| *Category* | *0* | *1–4* | *5–9* | *10–14* | *0–14* | *HV (%)* | *Crude rate* | *WASR* |
|---|---|---|---|---|---|---|---|---|
| *Males* | | | | | | | | |
| Ependymoma | 0 | 13 | 2 | 11 | 26 | 96.2 | 2.93 | 3.06 |
| Astrocytoma | 6 | 23 | 27 | 34 | 90 | 97.8 | 10.14 | 10.17 |
| Medulloblastoma | 2 | 29 | 27 | 26 | 84 | 97.6 | 9.47 | 9.74 |
| Other glioma | 0 | 5 | 18 | 9 | 32 | 18.8 | 3.61 | 3.51 |
| Miscellaneous intracranial and intraspinal | 0 | 4 | 12 | 7 | 23 | 56.5 | 2.59 | 2.52 |
| All CNS | 8 | 74 | 86 | 87 | 255 | 83.9 | 28.74 | 29.01 |
| *Females* | | | | | | | | |
| Ependymoma | 4 | 12 | 3 | 4 | 23 | 95.7 | 2.73 | 3.14 |
| Astrocytoma | 5 | 39 | 43 | 32 | 119 | 95.8 | 14.12 | 14.64 |
| Medulloblastoma | 2 | 10 | 27 | 11 | 50 | 94.0 | 5.93 | 5.99 |
| Other glioma | 1 | 6 | 13 | 9 | 29 | 13.8 | 3.44 | 3.41 |
| Miscellaneous intracranial and intraspinal | 1 | 0 | 7 | 10 | 18 | 83.3 | 2.14 | 1.91 |
| All CNS | 13 | 67 | 93 | 66 | 239 | 84.5 | 28.35 | 29.08 |
| *Males and females* | | | | | | | | |
| Ependymoma | 4 | 25 | 5 | 15 | 49 | 95.9 | 2.83 | 2.83 |
| Astrocytoma | 11 | 62 | 70 | 66 | 209 | 96.7 | 12.08 | 12.35 |
| Medulloblastoma | 4 | 39 | 54 | 37 | 134 | 96.3 | 7.74 | 7.91 |
| Other glioma | 1 | 11 | 31 | 18 | 61 | 16.4 | 3.53 | 3.46 |
| Miscellaneous intracranial and intraspinal | 1 | 4 | 19 | 17 | 41 | 68.3 | 2.37 | 2.22 |
| All CNS | 21 | 141 | 179 | 153 | 494 | 84.2 | 28.55 | 29.04 |

| HB | N | SRR | 95% | CI |
|---|---|---|---|---|
| A | 25 | 74 | 48 | 109 |
| B | 12 | 149 | 77 | 260 |
| C | 34 | 83 | 58 | 116 |
| F | 47 | 156 | 114 | 207 |
| G | 92 | 109 | 88 | 133 |
| H | 16 | 89 | 51 | 127 |
| L | 53 | 97 | 73 | 127 |
| N | 46 | 54 | 39 | 72 |
| S | 84 | 138 | 110 | 171 |
| T | 29 | 88 | 59 | 127 |
| V | 32 | 133 | 91 | 188 |
| Y | 17 | 132 | 77 | 211 |
| Z,R,W | 5 | 76 | 24 | 177 |

**Figure 2** Numbers of cases (*n*), standardised registration ratios (SRRs) of all CNS neoplasms for health board (HB) of residence for children 0–14 years, Scotland, 1975–90.

of cases points to inadequacies of the registration system and methods of case finding. This deficiency is currently being addressed, and use of computerised data from a variety of sources, including pathology departments, will assist in the future. The fact that the reviewing pathologists were the primary source of our additional cases appears to support this. Overall, the errors we have documented are substantial enough to influence the results of small-area geographical studies and underline the importance of validating data when such work is contemplated.

Descriptive epidemiology of childhood brain and CNS tumours, in common with all other childhood cancers, is based on a morphologically orientated classification scheme (Birch & Marsden, 1987), and statistics prepared on cancer registry data, and therefore coded only by site, may differ. The use of the Birch and Marsden scheme for our analyses gave us the most precise classification and facilitates comparison with other data sets. Overall, data for the two different classification schemes were numerically very similar and large-scale analyses would produce similar results from either data set. Pathological classification systems identify

**Figure 3** World age-standardised rates (per 1,000,000 population) for CNS and miscellaneous intracranial and intraspinal neoplasms by deprivation category for children 0–14 years, Scotland, 1975–90.

rare groups of tumours which may be aetiologically distinct but have to be subsumed into larger groups for descriptive analyses. The potential for trends in incidence of rare tumours to be masked by results from larger subgroups is always present. The scheme for classifying childhood brain tumours incorporates five subgroups, and inevitably specific unusual tumours are too few in numbers to be examined separately.

The age-standardised incidence rates for our validated Scottish data are higher (29.0 per million) than those previously published (21.2 per million) (Parkin *et al.*, 1988*a*), which may be accounted for either by previous underascertainment or by a rise in incidence. This higher rate puts Scotland close to the Scandinavian countries, which display the highest incidence in Europe: Sweden, 34.9 per million (Lannering *et al.*, 1990); Finland, 31.2 per million; and Denmark, 30.9 per million (Parkin *et al.*, 1988*a*). These are at least 50% higher than observed rates in Hungary (18.4 per million) (Parkin *et al.*, 1988*a*). Incidence in the north of England for an earlier time period (1968–82) gives a crude intermediate rate of 25.7 per million (Craft *et al.*, 1987). The relative frequency of the diagnostic subgroups reflects the pattern of other population-based studies (Parkin *et al.*, 1988*a*, Kallio *et al.*, 1991). The difficulties associated with the ascertainment of brain tumours and the fact that analysis of validated data gives rise to increased rates, as shown by our and other studies (Lannering *et al.*, 1990), suggests that published incidence figures based on cancer registration may be conservative. Sex ratios for the two most common subgroups of tumours, astrocytoma and medulloblastoma, show a female and a male excess respectively. For medulloblastomas this is consistent with international data (Parkin *et al.*, 1988*b*), but the finding for astrocytomas is unusual compared with other countries where sex ratios are generally close to unity. In the Scottish data the effect is most marked in the 1–9 year age group, however no explanation is immediately apparent for these observations.

Temporal trends in incidence for childhood brain tumours have received little attention in the literature, which makes comparison of the Scottish finding of a significant increase difficult to set in the context of other countries. In this group of tumours, changes in diagnostic practice over time have been substantial, and an increase in numbers could be explained by improved availability of biopsy material for histological diagnosis. The reasons for suggesting the rise is real rather than artefactual are 2-fold. Firstly, the proportion of 'other and unspecified tumours' has not decreased to the same extent over the time period (1975, 15.4%; 1990, 8.1%) and neither has the proportion of histologically verified tumours changed (1975, 84.6%; 1990, 91.9%). The particularly prominent increase in incidence is partially dependent on the low incidence rates in the early years of the study, however there is no evidence to suggest that ascertainment was especially poor in this early period.

Published scientific and medical literature does not appear to document geographical differences, particularly within a country, in incidence for this group of childhood malignancies. In Scotland significant variation exists by health board area, although the confidence intervals on the areas showing the significant excesses and deficits are wide. These results should be interpreted with caution. The methods used to optimise case ascertainment cross-checked sources which covered the whole of Scotland, and there is no evidence to suggest that levels of ascertainment differ by geographical region. The four neuropathology centres in Scotland receive cross-boundary referrals which do not necessarily relate to the proximity of the cases' residences at diagnosis. The reason for this geographical heterogeneity is difficult to explain in relation to environmental risk factors as so little analytical work has been done in this field. Visual inspection of the health board map of Scotland and accompanying SRRs (Figure 2) suggests a tendency for rural and sparsely populated area, particularly in the North, to have SRRs lower than 100. However, this, is not the case in the Borders and Dumfries and Galloway, where high SRRs are present. To confirm a tentative indication of increasing incidence moving from the north of Scotland to the south would require detailed areal studies adjusting for a variety of factors, including differentials in underlying incidence rates. The interpretation of our current observations is therefore limited.

Socioeconomic status and its relationship to childhood brain and CNS tumours and area of residence seems not to have been investigated elsewhere. Ecological correlation studies always have limitations in that they characterise an area and not an individual with the disease. A review of case–control studies investigating childhood brain tumours (Kuijten & Bunin 1993) reported two studies showing a positive association for parental 'professional occupations' and higher social class, which is consistent with the Scottish findings. Two further studies (Kuijten & Bunin, 1993) and one recent study (McCredie *et al.*, 1994) observed reduced levels of education in the parents of case children. The newly described association with higher social groups found in the present study requires confirmation from other independent data sets. However, the strength of the association means that future statistical analysis of distribution should account for socioeconomic status. The study findings of variation in incidence by both health board area and deprivation category appear to be independent of each other on preliminary examination of the data. Areas with a high proportion of post code sectors in the most affluent Carstairs category are not necessarily those with SRRs greater than 100. Similarly, those with high proportions of post code sectors in the most deprived category (e.g. Greater Glasgow) do not necessarily have low SRRs. The relationship between geographical area and socioeconomic level is currently being investigated.

In conclusion, we have demonstrated a requirement for validation of cancer registry data to be employed in detailed studies of geographical distribution where few cases can influence results. These types of studies should also consider taking socioeconomic levels into account.

We wish to thank the regional cancer registries in Scotland for their work contributing to the national data set, Dr J.A. Clarke for providing hospital discharge data and the Childhood Cancer Research Group at the University of Oxford for providing data from the UK National Registry of Childhood Tumours. We are particularly grateful to Mr C.A. Stiller, who kindly supplied a conversion program for the ICD and Birch and Marsden classification schemes. We are most grateful to Professor W.R. Lee, Professor J.H. Adams, Professor D.I. Graham, Dr J.M. Anderson (Dundee) and Dr P.J. Best (Aberdeen) and their secretarial and technical staff for invaluable assistance in the pathology review, and to Dr J.E. Bell (Edinburgh) for helpful discussion of many cases.

### References

BIRCH, J.M. & MARSDEN, H.B. (1987). A classification scheme for childhood cancer. *Int. J. Cancer*, **40**, 620–624.

BOYLE, P. & PARKIN, D.M. (1991). Statistical methods for registries. In *Cancer Registration Principles and Methods*, Jensen, O.M., Parkin, D.M., MacLennan, R., Muir, C.S. & Skeet, R.G. (eds). pp. 126–158. IARC Scientific Publications No. 95. IARC: Lyon.

BRESLOW, N.E. & DAY, N.E. (1987). *Statistical Methods in Cancer Research*. Vol. II, *The Design and Analysis of Cohort Studies*, IARC Scientific Publications No. 82. IARC: Lyon.

CARSTAIRS, V. & MORRIS, R. (1991). *Deprivation and Health in Scotland*. Aberdeen University press: Aberdeen.

CRAFT, A.W., AMINEDDINE, H.A., SCOTT, J.E.S. & WAGGET, J. (1987). The Northern region children's malignant disease registry 1968–82. *Br. J. Cancer.*, **56**, 853–858.

DAVIS, D.L., HOEL, D., PERCY, C., AHLBOM, A. & SCHWARTZ, J. (1990). Is brain cancer mortality increasing in industrial countries? *Ann. NY Acad. Sci.*, **609**, 191–204.

DRAPER, G.J., STILLER, C.A., FEARNLEY, H., LENNOX, E.L., ROBERTS, E.M. & SAUNDERS, B.M. (1988). United Kingdom England & Wales National Registry of Childhood Tumours 1971-90. In *International Incidence of Childhood Cancer*, Parkin, D.M., Stiller, C.A., Draper, G.J., Bieber, C.A., Terracini, B. & Young, J.L. (eds). pp. 295-304. IARC Scientific Publications No. 82. IARC: Lyon.

GLASS, S., GRAY, M., EDEN, O.B. & HANN, I. (1987). Scottish validation study of cancer registration data childhood leukaemia 1968-81. *Leuk. Res.*, **11**, 881-885.

KALLIO, M., SANKILA, R., JAASKELAINEN, J., KARJALAINEN, S. & HAKULINEN, T. (1991). A population-based study on the incidence and survival rates of 3857 glioma patients diagnosed from 1953 to 1984. *Cancer*, **68**, 1394-1400.

KLEIHUES, P., BURGER, P.C. & SCHEITHAUER, B.W. (eds) (1993). Histological typing of tumours of the central nervous system. In *International Histological Classification of Tumours*, 2nd edn, World Health Organization. Springer: Berlin.

KUIJTEN, R.R. & BUNIN, G.R. (1993). Risk factors for childhood brain tumours. *Cancer Epidemiol. Biomarkers Prev.*, **2**, 277-288.

LANNERING, B., MARKY, I. & NORDBORG, C. (1990). Brain tumours in childhood and adolescence in West Sweden 1970-1984. *Cancer*, **66**, 604-609.

LAPHAM, R. & WAUGH, N.R. (1992). An audit of the quality of cancer registration data. *Br. J. Cancer*, **66**, 552-554.

MCCREDIE, M., MAISONNEUVE, T., BOYLE, P. (1994). Ante-natal risk factors for malignant brain tumours in New South Wales children. *Int. J. Cancer*, **56**, 6-10.

MAO, Y., DESMEULES, M., SEMENCIW, R.M., HILL, G., GAUDETTE, L. & WIGLE, D.T. (1991). Increasing brain cancer rates in Canada. *Can. Med. Assoc. J.*, **145**, 1583-1591.

MUIR, C., WATERHOUSE, J., MACK, T., POWELL, J. & WHELAN, S. (eds) (1987). *Cancer Incidence in Five Continents*, Vol. V. IARC Scientific Publications No. 88. IARC: Lyon.

PARKIN, D.M., STILLER, C.A., DRAPER, G.J., BIEBER, C.A., TERRACINI, B. & YOUNG, J.L. (eds) (1988*a*). *International incidence of childhood cancer*. IARC Scientific Publications No. 87 IARC: Lyon.

PARKIN, D.M., STILLER, C.A., DRAPER, G.J., BIEBER, C.A. (1988*b*) The international incidence of childhood cancer. *Int. J. Cancer*, **42**, 511-520.

PERCY, C., VAN HOLTEN, V. & MUIR, C.S. (eds) (1990). *International Classification of Diseases for Oncology*, 2nd edn. World Health Organization: Geneva.

REGISTRAR GENERAL SCOTLAND (1975-90). *Annual Reports 1975-90*. HMSO: Edinburgh.

SCHOUTEN, L.J., JAGER, J.J. & VAN DEN BRANDT, P.A. (1993). Quality of cancer registry data: a comparison of data provided by clinicians with those of registration personnel. *Br. J. Cancer*, **68**, 974-977.

WORLD HEALTH ORGANIZATION (1976). *International Classification of Diseases for Oncology*. WHO: Geneva.

WORLD HEALTH ORGANIZATION (1977). *International Classification of Diseases, 1975 revision*. WHO: Geneva.

WORLD HEALTH ORGANIZATION (1992). *International Statistical Classification of Diseases and Related Health Problems*, Vol. 1, WHO: Geneva.